# Early life exposures of childhood asthma and allergies—an epidemiologic perspective

**DOI:** 10.3389/falgy.2024.1445207

**Published:** 2024-08-23

**Authors:** Rajesh Melaram

**Affiliations:** College of Nursing and Health Sciences, Texas A&M University—Corpus Christi, Corpus Christi, TX, United States

**Keywords:** early life, air pollution, pollen, asthma, allergies

## Abstract

Children around the world are continuing to develop and suffer from chronic lung diseases such as asthma. Childhood asthma commonly presents with recurrent episodes of cough, shortness of breath, and wheezing, all of which can lead to missed school days and hospitalization admissions. The role of environmental pollutants and aeroallergens has been increasingly recognized in relation to asthma etiology. We showcase the impacts of air pollution and pollen exposures in early life on childhood asthma and allergies through an epidemiologic perspective. We also examine the effects of indoor microbial exposures such as endotoxin and glucan on allergic diseases in schoolchildren as many spend most of their time in a household or classroom setting. Findings of this work can assist in the identification of key environmental factors in critical life periods and improve clinicians’ diagnoses of asthma during early childhood.

## Introduction

Asthma is a chronic respiratory disease caused by a combination of genetic, environmental, and lifestyle factors (1–3). Globally, asthma is the 28^th^ leading cause of disease burden and 16^th^ leading cause of years lived with disability, based on disability-adjusted life years. In the next year, the prevalence of asthma is expected to reach 400 million worldwide (4). Children represent a large fraction of asthma incidence and prevalence (5). Asthma development begins during early childhood and can progress throughout life, with some adults experiencing a first-time occurrence (4). Because of their underdeveloped organs and weakened immune systems, children are more susceptible to asthma symptoms (i.e., shortness of breath, cough, wheezing) and hospitalization admissions compared to their adult counterparts (6). However, asthma morbidity and mortality remain higher in adults (4) who have an increased risk of fixed airflow obstruction (7), alongside a greater potential for rapid lung function decline (4, 7, 8). Despite these differences, children often possess several risk factors for poor health outcomes and thus depend on others daily to meet their basic needs (9, 10).

Various environmental exposures are established risk factors for asthma including air pollution, tobacco smoke, and farm animals (11). The degree and timing of environmental exposures in early life (i.e., pregnancy and infancy) is crucial and may either serve as risk factors or protective factors for asthma. Maternal risk factors during pregnancy including antibiotic exposure, atopic disease, smoking, and stress have been associated with asthma (12, 13). Postnatal factors such as respiratory viral infections and indoor microbial exposures have shown to potentially safeguard against the development of asthma (12, 14). Climate change on the other hand is indirectly correlated with the increasing incidence of allergic diseases with recent focus on the role of air pollution and pollen exposures in asthma development and exacerbation (15). Studies provide compelling evidence that air pollution and pollen exposures result in asthma exacerbations and related allergy symptoms, as well as hospitalizations and emergency department presentations, respectively (16–20). The impact of prenatal and postnatal exposures to air pollution and pollen on childhood asthma and allergic disease risk is paramount and still uncertain in epidemiology.

As such, the primary objective of this perspective is to reveal the latest epidemiological evidence on early life exposures to air pollution and pollen exposures and their associations with childhood allergic diseases. In a secondary objective, we examine the role of microbial exposures on the risk of asthma and allergic conditions in schoolchildren. Enhanced knowledge of environmental exposures in early life can improve asthma prevention efforts, thereby improving the health of the pediatric population.

## Air pollution

### Particulate matter

Particulate matter (PM) consists of microscopic solid and liquid particles whose diameters are less than 10 (PM_10_) or 2.5 (PM_2.5_) micrometers. The impact of early life exposures of PM on childhood allergic diseases are well-studied (21–27) (Table 1). In a sex-stratified analysis, maternal daily exposure to elevated PM_2.5_ levels at midgestation (16–25 weeks) was found to associate with an increased risk of asthma development in urban boys by age 6 (21). Furthermore, a population-based study of four European birth cohorts reported an increased risk of asthma incidence after age 4 from early life exposure to PM_2.5_ (Odds Ratio [OR] 1.29, 95% Confidence Interval [CI] (1.00–1.66). The authors also examined the effect of PM_2.5_ on allergic rhinoconjunctivitis but did not identify a positive association (22). A Danish study on COPSAC_10_ birth cohort observed significant associations between postnatal PM_2.5_ [OR 1.51, 95% CI (1.08–2.07)] and PM_10_ [OR 1.56, 95% CI (1.14–2.09)] in relation to childhood asthma at age 6, supporting the impact of PM exposures during early life.

**Table 1:** Epidemiological studies on air pollution exposures in early life and childhood allergic diseases.

Pollutant	Location	Study design	Study population	Findings	References
Particulate matter 10 (PM_10_)	South Korea	Retrospective cohort study	Elementary schoolchildren (*n* = 3,570)	Airway hyperresponsiveness at age 1 potentially mediated prenatal PM_10_ exposure and childhood asthma at age 7	(23)
	China	Retrospective cohort study	Preschool children (*n* = 39,782)	PM_10_ exposure in early life associated with lifetime eczema	(27)
	China	Cross-sectional study	Young children (*n* = 29,418)	PM_10_ exposure in early life associated with childhood asthma	(25)
Particulate matter 2.5 (PM_2.5_)	United States	Retrospective cohort study	Full-term children (≥ 37 weeks, *n* = 736)	High prenatal PM_2.5_ exposure during midgestation increased the risk of asthma at age 6 in boys	(21)
	United States	Population-based cohort study	Young children (*n* = 1,469)	High prenatal PM exposure during the saccular phase (24–36 weeks gestation) increased the risk of asthma at age 4	(26)
	Brazil, Canada, China, Denmark, France, Norway, Poland, Singapore, South Korea, Spain, Sweden, United Kingdom, United States	Systematic review and meta-analysis	Young children (*n* = 133–93,635)	High prenatal PM_2.5_ exposure increased the risk of childhood asthma, atopic dermatitis, and hay fever	(24)
	Germany, Sweden, and Netherlands	Population-based cohort study	Participants from four prospective birth cohorts (*n* = 14,126)	PM_2.5_ exposure in early life increased the risk of asthma development after age 4 but not rhinoconjunctivitis	(22)
Nitrogen dioxide (NO_2_)	Canada	Population-based nested case-control study	Resident children (*n* = 37,401)	High prenatal and postnatal NO_2_ exposures increased the risk of asthma in preschool children	(34)
	Brazil, Canada, China, Denmark, France, Norway, Poland, Singapore, South Korea, Spain, Sweden, United Kingdom, United States	Systematic review and meta-analysis	Young children (*n* = 133–93,635)	High prenatal NO_2_ exposure increased the risk of childhood asthma, atopic dermatitis, and hay fever	(24)
	China	Retrospective cohort study	Preschool children (*n* = 3,358)	NO_2_ exposure in the first year of life was associated with an elevated risk of asthma and rhinitis in preschool children	(41)
	United States	Prospective cohort study	Pediatric primary care patients (*n* = 947)	Exposure to indoor and outdoor NO_2_ in rural-urban settings was not associated with childhood asthma	(30)
Sulfur dioxide (SO_2_)	Canada	Population-based cohort study	Young children (*n* = 722,667)	Residential exposure to elevated SO_2_ emissions increased the risk of asthma development in children before age 4	(31)
	Canada	Population-based nested case-control study	Residential children (*n* = 37,401)	Prenatal and postnatal SO_2_ exposures increased the risk of asthma development in preschool children	(28)
	China	Retrospective cohort study	Preschool children (*n* = 3,358)	Prenatal SO_2_ exposures was not associated with childhood asthma and rhinitis	(29)
Ozone (O_3_)	Canada	Administrative cohort study	Residential children (*n* = 1,183,865)	Residential exposure to elevated O_3_ levels at birth increased the risk of childhood asthma	(33)
	Canada	Population-based cohort study	Schoolchildren (*n* = 1,286)	Exposure to O_3_ at birth increased the risk of allergic rhinitis and eczema	(34)
	China	Case-crossover study	Children with asthma attacks (*n* = 3,714)	Increased O_3_ levels at low concentrations increased the risk of asthma attacks	(32)

Location and study population for systematic review and meta-analysis study includes more than one country and sample size range for all eligible included studies.

In terms of childhood allergic diseases, there are fewer studies addressing the effect of prenatal PM exposures, particularly on asthma. In the United States, a multicity sample of two pregnancy cohorts demonstrated the saccular phase (24–36 weeks gestation) to be a critical window for PM_2.5_ exposure and subsequent development of asthma at age 4 (27). A mediation analysis on prenatal PM_10_ exposure and asthma incidence showed that airway hyperresponsiveness at age 1 potentially mediates the association between exposure to PM_10_ during the second trimester and asthma incidence at age 7 in schoolchildren (23). For early life exposure, outdoor PM_10_ was found to associate with lifetime eczema [OR 1.17, 95% CI (1.06–1.28)], but not asthma, wheeze, or rhinitis among preschool children (26). A different study observed an increased risk of childhood asthma from early life exposure to PM_10_ [OR 1.11, 95% CI (1.02–1.20)]. Compared to asthma, more attention has shifted towards allergic rhinitis as of late given its increased prevalence alongside the compounding effects of climate change and ambient particle pollution in Westernized countries.

### Nitrogen dioxide

Nitrogen dioxide (NO_2_) is a gaseous oxide commonly produced by combustion processes, fossil fuel emissions, industrial activities, and transportation (35). Indoor sources of NO_2_ include building heat, natural gas stoves, and tobacco smoke (36, 37). Because children spend ∼70% of their time inside, they may be exposed to higher levels of indoor NO_2_ than outdoor NO_2_ (38). Epidemiological studies have identified associations between outdoor NO_2_ exposure and risk of childhood asthma and wheezing (39, 40). The effect of prenatal and postnatal NO_2_ exposures on childhood asthma remains less investigated, with few studies highlighting an increased risk of asthma in preschool-aged children (28, 29). More recently, a prospective cohort study explored the relationship of indoor and outdoor NO_2_ in mixed rural-urban settings with childhood asthma; however, no significant association was detected (30). The feasibility study used a small sample (*n* = 947), which may have contributed to a lack of an association.

Moreover, a study in Canada explored the role of NO_2_ exposure in the first year of life and risk of allergic diseases including asthma, allergic rhinitis, and eczema. Early life exposures to NO_2_ were associated with an increased risk of incident asthma [OR 1.06, 95% CI (0.96–1.16)] and eczema [OR 1.05, 95% CI (0.99–1.11)] in comparison to allergic rhinitis [OR 0.94, 95% CI (0.87–1.02)] (34). In China, a group of researchers analyzed the association between prenatal and postnatal NO_2_ exposures and childhood allergic rhinitis prevalence. No significant association was observed for prenatal NO_2_, while an increased odds of allergic rhinitis resulted from postnatal exposure in the first year of life [OR 1.013, 95% CI (1.002–1.025)] (41). Considering these findings, the association between early life exposure to NO_2_ and childhood allergic diseases are inconsistent and necessitate longitudinal cohort studies.

### Sulfur dioxide

Sulfur dioxide (SO_2_) is a noxious gas primarily generated from fossil fuel combustion or industrial processes. Epidemiological studies revealed an increased risk of asthma exacerbations in children exposed to high levels of SO_2_ in the short term (42–44). Continued interest in this arena may be due to the toxic effects of SO_2_ in sensitive asthmatics, although the exact mechanisms are not completely understood (45). However, researchers report inconsistent findings on early life SO_2_ exposures and risk of asthma and allergic rhinitis. For example, residential exposure to industrial SO_2_ emissions in Quebec, Canada was shown to contribute to childhood asthma development mostly before 4 years of age (31). In an earlier study, children exposed to high levels of SO_2_ in pregnancy and in infancy had an elevated risk of asthma onset [OR 1.03, 95% CI (1.02–1.05) for both periods] (28). No significant relationship was observed between prenatal SO_2_ exposure and risk of childhood asthma and allergic rhinitis in China (29). A follow-up study among Korean schoolchildren discovered a high risk of allergic rhinitis associated with high atmospheric SO_2_ concentrations [OR 1.056, 95% CI (1.006–1.109)] (46). With continued investigations on SO_2_ exposures and childhood allergic diseases associations may become clearer.

### Ozone

Ozone (O_3_) is a reactive gas existing in both the stratosphere and troposphere of Earth's atmosphere. Stratospheric O_3_ prevents harmful health effects by absorbing ultraviolet rays from the sun. Inhalation of tropospheric O_3_ at high concentrations can cause cardiovascular and respiratory diseases in children (47). There is increasing evidence supporting the relationship of O_3_ exposure with asthma exacerbations or development in childhood. A case-cross over study found that O_3_ exposure >80 µg/m^3^ increased the risk for asthma attacks on each day of lag, with a significant effect observed for levels >100 µg/m^3^ (32). Conversely, findings from an administrative cohort study in Québec, Canada indicated an increased risk of asthma development with respect to residential exposure to O_3_ at birth [Hazard Ratio (HR) 1.11 95% CI (1.10–1.12)] (33). Associations have similarly been reported for O_3_ exposure at birth and childhood allergic rhinitis [HR 1.15, 95% CI (1.00–1.31)] and eczema [HR 1.05, 95% CI (0.95–1.16)] (34).

## Pollen

With global climate change extending the pollen season and distribution of airborne pollen, the adverse effects of early life pollen exposures on young children are becoming more pronounced. Several factors contribute to variable pollen levels in the atmosphere including vegetation source (i.e., trees, grasses, and weeds), seasonality, and weather conditions (48). Seasonal pollen exposure stimulates IgE-mediated inflammatory responses, resulting in itchy eyes, nasal congestion, rhinorrhea, and persistent sneezing (49, 50). Many epidemiological studies have thus investigated associations between pollen season of birth and childhood allergic diseases (51–53). Given the increasing trends in global climatic patterns some studies are seeking to understand the impact of pollen exposures on asthma and allergic disease outcomes (Table 2). For instance, one study reported an increased risk of sensitization to atopic disease from high pollen exposure during pregnancy and in infancy, with the latter showing a greater tendency towards sensitization (55). An earlier cross-sectional study by the same authors revealed an increased odds of sensitization [OR 2.4, 95% CI (1.2–4.6)] and allergic asthma [OR 2.6, 95% CI (1.2–5.6)] from high pollen exposure in infancy.

**Table 2:** Epidemiological studies on pollen exposures in early life and childhood allergic diseases.

Location	Study design	Study population	Findings	References
Sweden	Cross-sectional study	Children with atopic heredity (*n* = 583)	High levels of exposure to birch pollen during infancy increased the risk of atopic disease in children aged 4–5 years	(55)
Sweden	Cross-sectional study	Children identified from birth records (*n* = 1,725)	High levels of exposure to birth pollen during infancy exhibited a greater impact on the risk of atopic sensitization in children aged 4–5 years compared to high levels of the same allergen during pregnancy	(54)
Sweden	Register based cohort study	Singleton children delivered vaginally (*n* = 110, 381)	Prenatal and postnatal pollen exposures associated with an increased risk of asthma hospitalizations during infancy	(20)
Australia, Canada, Israel, United States, Spain	Systematic review and meta-analysis	Young children and adolescents (*n* = 1,076–199,533)	Exposure to grass pollen associated with childhood asthma emergency department presentations	(19)
Australia, Austria, Denmark, Finland, France, Germany, Spain, Switzerland, United Kingdom, United States	Systematic review and meta-analysis	Children and adults (*n* = 12–430)	Acute exposure to grass pollen associated with an increased risk of allergy and asthma symptoms	(56)

Location and study population for systematic review and meta-analysis studies includes more than one country and sample size range for all eligible included studies.

Another study noted an increased risk of asthma hospitalization from pollen exposure during late pregnancy and in the first year of life (20). Interestingly, the same study showed that high pollen levels in early pregnancy had a protective effect on asthma hospitalization by age 1. Limitations within the study included a lack of allergic disease phenotypes and potential misclassification of pollen exposures (20).

## Microbial allergens

### Bacterial endotoxin

Bacterial endotoxins are lipopolysaccharide (LPS) molecules found in Gram-negative bacteria. Multiple studies from the early 2000 s assessed the relationship between household endotoxin exposures and allergic diseases among schoolchildren (57–61). Various associations on endotoxin exposure occurred from these studies including an increased or a decreased risk of asthma or allergies to no association with asthma. In a longitudinal study of children with a parental history of atopy, high endotoxin exposure at ages 2–3 months associated with a decreased odds of atopy [OR 0.6, 95% CI (0.3–1.4)] and rhinitis [OR 0.3, 95% CI (0.1–0.9)] in schoolchildren, but an increased risk of wheeze from ages 1 to 7 years [HR 1.23, 95% CI (1.07–1.43)]. No significant association occurred between endotoxin exposure and asthma at 7 years (62). A case-control study in Canada suggested an increased risk of asthma or wheeze at age 12 in children whose parents reported a history of allergic disease to endotoxin exposure, but not for non-allergic children (63). These findings demonstrate that parental history of atopic disease could play a part in the protective effect of high postnatal endotoxin exposure and development of allergic diseases at earlier ages, and thus be treated as a potential confounder in epidemiological analyses.

### Glucan

Fungi are heterotrophic microorganisms composed of chitinous cell walls and release spores for dispersal and colonization. Few exposure assessments on fungal species or glucan in house dust have been conducted (64–66). High glucan exposure has been associated with a decreased risk of atopic eczema by school age [OR 0.73, 95% CI (0.51–1.05)] (67). Similarly, a decreased risk of allergic sensitization in children ages 2–4 years resulted from glucan exposure [OR 0.67, 95% CI (0.56–0.81)] (68). In Puerto Rico, an increased odds of atopy in control subjects [OR 2.46, 95% CI (0.37–4.55)] and emergency department/urgent care visits for asthma [OR 8.76, 95% CI (2.70–28.4)] was reported in schoolchildren exposed to high glucan levels (69). The study was cross-sectional in design, meaning it only captured the link between glucan exposure and risk of atopy and emergency department visits at a single time point. Collectively, epidemiological studies on glucan exposure are scarce, suggesting mixed findings on the risk of atopic and allergic phenotypes. Longitudinal cohort studies are therefore needed to assess the temporal sequence of indoor fungal exposures and allergic diseases in schoolchildren.

## Conclusion

This perspective sheds light on air pollution and pollen exposures as key environmental determinants in early life with respect to childhood allergic diseases. Findings on prenatal and postnatal air pollution and pollen exposures were analyzed and presented to indicate the potential risk of developing asthma and allergy in early childhood. As climate change continues to influence environmental changes, the interactive effects of air pollution and pollen exposures on allergic diseases may receive increasing attention. We even evaluated the role of indoor microbial exposures in connection with allergic diseases in schoolchildren. Despite conflicting findings on postnatal endotoxin and glucan exposures, future studies should explore maternal exposure to indoor pollutants and aeroallergens as well as potential mediators (i.e., viral infection and DNA methylation) with childhood allergic diseases.

## Data Availability

The original contributions presented in the study are included in the article/Supplementary Material, further inquiries can be directed to the corresponding author.
